# Children’s Olfactory Picturebooks: Charting New Trends in Early Childhood Education

**DOI:** 10.1007/s10643-023-01457-z

**Published:** 2023-03-18

**Authors:** Natalia Ingebretsen Kucirkova, Selim Tosun

**Affiliations:** 1 University of Stavanger, Hulda Gaborgs Hus, 4320 Stavanger, Norway; 2 Ankara University, Graduate School of Health Sciences, 06110 Ankara, Turkey

**Keywords:** Picturebook, Reading, Literacy, Olfaction, Smell

## Abstract

Converging global trends (digitization, globalization, datafication) have influenced all aspects of children’s literacies, including children’s picturebooks. The recent turn towards embodied, affective and sensory literacies, stimulated our interest in multisensory picturebooks that engage all children’s senses, including the sense of smell (olfaction). Olfactory children’s picturebooks demand new forms of literary conversations, which capitalise on unique properties of odours and integrate these with stories. Drawing on a systematic search of children’s picturebooks about, and with, smell, in paper-based and digital formats, we identified three principal ways in which olfaction is currently embedded in children’s picturebooks: 1, as an add-on to depiction of objects (including foods, plants) and places, 2, as a device to introduce humour into a story, and 3, as an engagement tool for children’s active participation in the story. We mobilise Sipe’s (2008) concept of seven constituting elements in children’s picturebooks to describe how current olfactory picturebooks apply the elements in their design and make recommendations for future development of children’s olfactory picturebooks. Reflecting on the generative potential of literary theories and olfactory power to stimulate children’s non-linguistic embodied interactions with picturebooks, we propose some extensions to the current olfactory picturebook landscape.

## Introduction

Given the vital role that stories play in structuring our thoughts and experiences, it is not surprising that the study of children’s picturebooks is such a vibrant research field. Researchers have illuminated the paramount role reading picturebooks plays in children’s language (Ommundsen et al., 2021) and socio-emotional development (e.g., Nikolajeva, 2017; Gunn et al., 2022). What is of particular research interest are the ways in which picturebooks communicate cultural and social meanings (Coats, 2017) and how these evolve with new means of communication and literacies. The recent turn towards phenomenological (Rowsell, 2014), affective (Leander & Ehret, 2019), and embodied (Hillesund et al., 2022) literacy studies with adult readers have also influenced literary understandings of children’s picturebooks (Papen & Peach, 2021; Zapata, 2022). The phenomenological turn inspired our thinking around the body-mind connection in children’s literacy. Rooted in the theoretical scholarship of Merleau-Ponty (2013), Rowsell (2014) highlighted the notion of the whole body, which through its movement and being in the space, acts as the source of perception. The affective turn, with its emphasis on the emergent learning that happens through bringing people together in an assemblage (Lenters, 2016), guided our thinking about the unfolding nature of children’s literate becomings. The phenomenological and affective turns are closely related to the embodied orientation towards literacy, which encompasses the assumption that multisensory, cognitive and tactile responses are enmeshed with the materiality of reading, and in our case, represented by olfactory picturebooks.

Our work connects to this line of research with a particular focus on children’s literary understandings stimulated with “hidden”, or less researched senses, particularly the sense of smell (olfaction). Recent advancements in olfactory technologies and public interest in the power of the sense of smell following COVID-19 disease, as well as the theoretical preoccupations with sensory, embodied and affective reading engagements, offer fertile ground for new research on children’s olfactory picturebooks. The recent COVID-19 induced anosmia (loss of sense of smell) and smell dysfunction as among the most common symptoms of the disease experienced by millions of people globally, boosted the interest in the sense of smell and resources that can train and stimulate a healthy sense of smell from young age.

In exploring the olfactory potential in children’s picturebooks, we systematically analyse the extent to which popular English children’s picturebooks directly refer to, or physically stimulate, children’s sense of smell. We take, as the point of departure, Sipe’s (2001) seminal work on social construction in literary understanding, and take a reflective and generative outlook on the ways in which a focus on olfaction might offer new insights into children’s picturebook studies and development. Our aim is to open new vistas for children’s literary studies and stimulate innovative research-driven developments in the production of children’s literature.

## Definition of Key Terms

Sipe (2008) makes it clear at the outset of his writings that picturebooks are not illustrated books or books with pictures. Rather, picturebooks are books and pictures merged together. The term picturebooks, as one word, reflects the intention to refer to these literary objects as one resource where words and images become intimately connected and interwoven in a double-act of fostering children’s meaning-making. We thus use the term “picturebooks” and define it as ’unique visual and literary art form that engages young readers and older readers in many levels of learning and pleasure’ (p. 273, Driggs Wolfenbarger & Sipe, 2007). We are interested in the most popular picturebooks and gauge their popularity by focusing on titles that are most often bought, ranked high, reviewed, or commented on the Amazon marketplace for children’s English books.

Another key term that needs to be clarified is “olfaction”. Olfaction refers to children’s sense of smell that is connected to, but physiologically and conceptually different from, the sense of taste. The human nose can detect one trillion odours (Bushdid et al., 2014), and these are processed in the limbic area of the brain (Herz & Engen, 1996). Olfactory perception involves complex emotion and memory processing, which are non-verbal and non-linguistic. While a few indigenous languages, for example the Austroasiatic languages and particularly the Jahai language (Burenhult & Majid, 2011), contain several abstract descriptive odour categories, most actively spoken languages have only a dozen words to capture various odours, aromas, and fragrances around us.

Our positionality as two researchers based in different countries, each with different traditions of children’s literature, is that children’s picturebooks carry profound learning, cultural and identity-related values, and that new ways of reading, such as those afforded by olfactory books, can enrich children’s literature in any context. The theoretical framework guiding our thinking in the analysis and interpretation of our findings is that of Sipe (2008) and the concept of a literary conversation guide, which synthesises insights from semiotics, visual aesthetic theory, schema, and cognitive flexibility theories.

The recent global pandemic and the loss of olfaction among COVID-19 sufferers has renewed the public interest in the importance of the human sense of smell (Aziz et al., 2021). Our interest in olfaction is a partial response to this interest, although we are interested not in what gets lost but what could be gained if we turned a critical lens on children’s healthy sense of smell and consider its potential for children’s picturebooks.

## The Power of Olfaction for Children’s Reading

Olfaction and stories are closely related in that humans have shared stories since prehistoric times, and olfaction, is considered the oldest communication form (Hoover, 2010). Olfaction has always been part of story-sharing in the form of ambient odours in the environment, odours inside reading substrates, and body odours of readers and story-tellers. The role that olfaction plays in stories is, however, very little researched. The Proust effect, which refers to the activation of autobiographical memories, has been described anecdotally in several novels, and appraised for the olfactory power in eliciting memories that may have been unattainable through other senses (Chu & Downes, 2000).

The principal brain part responsible for acquiring, consolidating, and retaining olfactory emotions is the amygdala (Menini, 2009), a small subcortical structure, located in each hemisphere of the brain. Neuroscientific studies have documented higher amygdala activation when processing memories (Granger & Lynch, 1991; Herz & Engen, 1996) and when processing emotional and pleasure-oriented olfactory information (Zald & Pardo, 1997). What is crucial for our focus here is that the close connection between olfaction, memory, and emotions can be harnessed for learning effects. Recent studies show that, for example, including rosemary, lemon, and peppermint stimulates students’ moods and higher learning performance (Choi et al., 2022). Despite the well-documented role of olfaction in memory and emotion studies, and its emerging potential role in learning, the sense of smell has been little studied in early childhood studies. Olfaction research has been the domain of consumer research, in particular food studies (Spence, 2015) and art and entertainment interventions (see Spence, 2020). With children, olfactory research focus has been limited to a clinical perspective (e.g., Gellrich et al., 2021).

Recently, however, the embodied and affective turn in children’s literacies (see Mills et al., 2022) has prompted a new attentiveness to children’s learning through and with all senses, including smell. Sensory reading (Kucirkova, 2022) is concerned with inter- and intra-action of all six senses in reading (vision, hearing, touch, smell, taste, and proprioception). In sensory reading, scents and smells are considered material qualities of picturebooks that together with other modes, such as music in digital picturebooks or texture in print picturebooks, influence the aesthetic qualities of a reading experience (Kucirkova, 2022).

The materiality of paper and digital books varies, and this is reflected in how aesthetically pleasing they are (Sipe, 2000, 2001). Paper-based picturebooks have material aesthetic markers such as size, cover, binding, and choice of paper, while digital books have materially ambiguous aesthetic markers such as hotspots, menu displays, and text placements (Al-Yagout & Nikolajeva, 2017). Scent can be added to both print and digital picturebooks in both material (e.g., scratch and sniff surface) and immaterial forms (e.g., textual description of a passage invoking one’s olfactory memory).

If we consider texts and images as joint contributors to meaning-making with metaphors and symbolism embedded in picturebooks (see Kress & van Leeuwen, 2020), then we can consider scent as a meaning-making mode that may reveal previously hidden relationships between other modes, for example, the communicative functions of pictures and texts. In this paper we examine this proposition with attention to the literary qualities of picturebooks that contain a verbal or physical reference to olfaction.

## The Present Study

In order to understand what a focus on the sense of smell can offer the children’s picturebook, we need to consider both material and immaterial forms of olfaction. We begin this consideration by first mapping the territory of children’s picturebooks *about* smell. We then review olfactory children’s picturebooks *with* smell and available in print or digital formats (as a prototype or commercial form). Taking these insights together, we discuss the added value of olfaction to children’s picturebooks now and in the near future. Our discussion is guided by Sipe’s (2008) analytical framework of literary conversation guides and the theoretical insights of embodied and affective literacy studies (Ehret & Rowsell, 2021; Lee et al., 2022).

We aim to find out:

How do current popular children’s picturebooks refer to children’s sense of smell?

How can smell enrich children’s picturebooks in the future?

## Methods

### Children’s Picturebooks About Smell

In order to gauge the popularity of children’s olfactory picturebooks, we followed the method used by other researchers (see, for example, Hu et al., 2022; Richardson et al., 2008) of searching the Amazon bestselling lists. We systematically searched the Amazon list of children’s books focused on smell and scents either in their title or book description, using the following keywords: “smell”, “scent”, “odour”, “perfume”, “fragrance”. The search was performed on the 23rd of September 2022, and was limited to the 3-5-years age range. We took into account both the results generated by the search and Amazon’s automatically generated list of “recommended titles”. The search generated 75 results pages, with a total of 1200 books. The whole list was formed with the “web scraping” method and the titles of the books were manually checked one by one. Relevant titles were entered into an Excel sheet. Out of the 1200 books, 102 books were considered relevant and retained. These books were looked up on their Amazon selling page or, in the case of insufficient information, on another publisher’s or selling platform. A simple content analysis was performed of each title with attention to how olfaction was used in the book by both authors. Discrepancies were discussed and consensus reached through discussion, as per standard qualitative content analyses (Drisko & Maschi, 2016).

## Findings

### Children’s Picturebooks *About* Smell

Our content analysis of 102 olfaction-related picturebooks resulted in five main categories of olfactory references: flower-related, food-related, related to bad smells, related to either potty training or to other senses (multisensorial, for example smell and vision indicating that something nice smells nice). Each book was placed in one of the five categories and the number of books in each was counted. The results are summarised in Table 1.

**Table 1:** Olfaction-related picture books with categories and examples

Category	N	Examples of book titles
Flowers	3	Chrysanthemum: A First Day of School Book for Kids by Kevin Henkes Magnolia Flower by Zora Neale Hurston, Ibram X. Kendi, et al. I’m a Flower Girl! Activity and Sticker Book (Bloomsbury Activity Books) by Bloomsbury Publishing PLC and Claire Keay
Food	74	Ice Cream Soup (Step into Reading) Part of: Penguin Young Readers, Level 1 (20 books) by Ann Ingalls Creepy Carrots! (Creepy Tales!) by Book 1 of 3: Creepy Tales! by Aaron Reynolds and Peter Brown The Happy Pumpkin (First Seasonal Stories) Green Eggs and Ham Part of: First Seasonal Stories (3 books) by DK and MacKenzie Haley
Other senses	5	See, Touch, Feel: A First Sensory Book by Roger Priddy The Feelings Book by Todd Parr My Five Senses (Let’s-Read-and-Find-Out Science 1) by Aliki
Bad odours	7	I Love You, Stinky Face by Lisa McCourt and Cyd Moore ; The Stinky Cheese Man and Other Fairly Stupid Tales Part of: Caldecott Honor Book (5 books) by Jon Scieszka and Lane Smith Walter the Farting Dog Part of: Walter the Farting Dog (6 books) by William Kotzwinkle, Glenn Murray, et al.
Potty training	13	Let’s Go to the Potty!: A Potty Training Book for Toddlers by by Allison Jandu Everyone Poops (Taro Gomi by Chronicle Books) by Taro Gomi Daniel Tiger’s Potty Time! Children’s Toilet Training Sound Book for Daniel Tiger Fans (Daniel Tiger Neighborhood) by Scarlett Wing and Cottage Door Press

The content analysis showed that the use of olfaction in the most sold (or popular) children’s picturebooks on the global, English-speaking, Amazon marketplace, is mostly in relation to food and bad odours. The latter is mostly used in humour-oriented stories or toddler’s books about potty training. Very few books focus on the pleasant smells of flowers (three) or smell related to other senses (five). To survey the current offer of digital olfactory picturebooks, we conducted the same keyword search in the App store on Apple and GooglePlay (children’s category). This did not return any relevant children’s apps or e-books that were about smell.

### Children’s Picturebooks *with* Smell

In our analysis of olfactory books, we considered both print and digital picturebooks. For print picturebooks, we searched the global Amazon children’s booklist with the keyword “scratch and sniff”. This generated 198 results, which we manually searched for relevance, focusing on, for example, whether they contained literary qualities that would qualify them as picturebooks and not illustrated books, and whether the scratch and sniff technology was embedded inside the books to release various odours. We excluded books that contained scratch and sniff technology as an activity (e.g., *Scratch and Sniff: Halloween* (DK Publishing, 2001)) rather than as part of a story. In total, 87 titles were relevant, and these were analysed for main topics dealt with in the books. Table 2 summarises the results.

**Table 2:** Olfaction-related books with scratch and sniff surfaces with examples and categories

Topics	N	Examples of book titles
Flowers	5	*Ice Age: Stop and Smell the Dandelion* (Teitelbaum, 2002) *Sesame Street: Holiday Friends* (Mitter, 2017) *Scratch & Sniff Floral Fairies* (Melissa & Doug, 2017)
Food	33	*Peppa Pig: Peppa Loves Fruit Scratch & Sniff Sound Book* (PI Kids, 2023) *My First Games Readers: Sweets and Treats* (Hughes, 2001) *Instant Touch: A Tropical Scratch and Sniff* (Belly Kids, 2014)
Other senses	7	*Follow Your Nose! A Scratch-and-Sniff Adventure* (Random House, 2018) *Clifford Follows His Nose* (Bridwell, 1992) *Peter Follows His Nose: A Scratch-and-Sniff Book* (Potter, 2019)
Bad smells	3	*This Book Stinks!* (Webster, 2019) *Pirate Pete and His Smelly Feet* (Rowland, 2017) *Color Dog* (Fleet, 2015)
Festivities	32	*A Candy Christmas Scratch and Sniff Sticker Book to Color* (Dalmatian Press, 2008) *Santa’s Biggest Little Helper (Golden Scratch & Sniff Book)* (Dubowski, 1997) *A Scratch & Sniff Halloween* (Spurr, 2009)
Abstract scents	5	*The Sweet Smells of Zahramay Falls* (Tillworth, 2018) *The Smell of a Rainbow* (Goldworm, 2021) *Tinker Bell’s Scratch and Sniff Surprises* (Posner-Sanchez, 2009)

Scratch and sniff books are books with selected scratchable areas, which, when rubbed, activate the release of an odour. Scratch and sniff books have enjoyed great popularity since their first release in the 1980s, and they are, according to our search results, still popular among readers. Examples of the most popular titles include the *Scratch And Sniff: Food* (DK Publishing, 1999); *Follow Your Nose, Everyday Scents* (Auzou, 2020), and *Llama Llama Yum Yum Yum!: A Scratch-and-Sniff Book* (Dewdney, 2016). While in these titles, smell is the leading theme, in other titles, smell was added to a popular existing story. For example, in the scratch and sniff version of *The Very Hungry Caterpillar’s Garden Picnic* (Carle, 2020), smell was added to the existing well-known story. Our analysis also revealed that a sizeable proportion of scratch and sniff books are dedicated to the food and festivities themes, presumably given the interactive nature of festivities and the close connection between food and smell.

The number of smells (scratchable surfaces) in the reviewed books ranged from five to seven per book. Some smells were used to represent places (e.g., in the book *Follow Your Nose, Everyday Scents* (Auzou, 2020), smells refer to 7 different everyday scenes: in the garden, in the forest, at the market, in the kitchen, at the funfair, and in the country), while in other titles, the odours relate to various foods such as pickles and ice-cream in *Lama Llama Loves His Mama* (Dewdney, 2021). We tested the Norwegian version of the scratch and sniff book *Peter Follows His Nose* (Potter, 2019), which contains five smells related to five foods or plants that Peter the Rabbit finds on his adventure, including: strawberry, mushrooms, lavender, peppermint, and blueberry cake. The references to the smell pages are made clear in the text, with a simple storyline that revolves around the various smells Peter encounters on his way home.

In our experience of reading this book with Norwegian families, the match between the olfactory reference and actual smelled fragrance was very low. Most families reported the smell was either non-existent on the page or different from its textual reference. This experience mirrors that of thousands of low or negative ratings on Amazon regarding the books’ olfactory qualities. A quick perusal of customers’ reviews reveals discontent with the intensity and duration of the individual smells and their authenticity in representing the objects they are associated with in the books. The short duration and difficulty of representing concrete odours through the scratch and sniff technology is well-known in the publishing world (Aschehoug publishing house, personal communication).

As for *digital* children’s books with smell, we conducted a Google search using the keywords children, digital, olfactory/smell/scent/fragrance/odour [alternated], children/kids [alternated]. The search revealed that, thus far, the only digital picturebook that integrated olfaction with a digital children’s picturebook was the ‘oBook’, released in 2014. The prototype was part of a research project carried by Professor David Edwards with his students Rachel Field and Amy Yin. The picturebook was digital and presented on an iPad, with several hotspots, which, when touched on the screen, released a smell from a Bluetooth-connected aroma dispenser connected to the tablet. Obooks are an innovative and disruptive concept that challenges the status of interactions between science, technology, and education. Olfactory mechanisms used in art, museum, and entertainment contexts include fragrant technology, aroma dispensers, or specially designed aromatic cartridges. Some are stationary and some are portable, such as the Aromastic technology developed by SONY. These mechanisms could be usefully deployed for children’s digital picturebooks in the future.

To stimulate future research and design in the area, we discuss the current trends of olfactory books in relation to future directions in children’s literary works.

## Discussion

Our discussion is guided by Sipe’s (2008) concept of a literary conversation guide, which draws on both children’s responses to picturebooks and insights from semiotics, visual aesthetic theory, schema, and cognitive flexibility theories. The reason we adapted literary conversation guides for our consideration of future children’s olfactory picturebooks is that the concept provides a framework for matching established conventions in children’s picturebooks with innovative possibilities.

Sipe (2008) proposed seven elements in children’s picturebooks that have a semiotic (meaning-making) significance: Speculating on actions the characters might have performed; Creating imaginary dialogue that might have occurred among characters; Creating possible thoughts and feelings of characters; Talking about likely changes of setting between page breaks; Speculating on the amount of time that might have elapsed between one page opening and the next; Hypothesizing about the changes in the reader’s perspective; Changes from realistic fiction to fantasy. We applied these seven elements to our integrative interpretation of the current, and possible future, design of children’s olfactory picturebooks. We present the seven elements with our reflections on how they correspond to current and future olfactory picturebooks. We summarise our key considerations in Table 3 in relation to current and possible future olfactory picturebooks and we synthesise the insights in the formulation of seven guiding principles for early childhood researchers and picturebook publishers.

**Table 3:** Current and future olfactory picturebooks according to guiding literary conversation principles

Literary conversation guide according to Sipe (2008)	Current olfactory picturebooks	Future olfactory picturebooks
Speculating on actions the characters might have performed	Dominance of concrete smells that are linked to characters’ actions described in text and illustrations	Implied actions that are less concrete and more open to children’s inferences
Creating imaginary dialogue that might have occurred among characters	Imaginary dialogue was not represented in any of the resources reviewed	The use of ambient scents
Creating possible thoughts and feelings of characters	Individual characters carried concrete, mostly unpleasant, smells intended to produce humour	Odours can convey various and complex personality traits
Talking about likely changes of setting between page breaks	Scratch and sniff areas are presented in a fixed sequence	Essences could be used to create and advance scenes in various directions
Speculating on the amount of time that might have elapsed between one page opening and the next	Scratch and sniff books allow for calibrating odour intensity to a certain extent in that readers can scratch more or less using their fingers	Odour intensity thresholds could be calibrated with bespoke fragrances or custom scented aromas that are made available to readers
Hypothesizing about the changes in the reader’s perspective	Current examples use olfaction to refer to basic emotions e.g., surprise, or confirmation of perception e.g., disgust at bad smell.	Scents could qualitatively enrich the story by increasing readers’ empathy with the story characters through conveying more clues about the character’s environment and feelings
Changes from realistic fiction to fantasy	The reviewed picturebooks focused on concrete smells and realistic fiction	Transitions from fictional or fantasy worlds can be facilitated with changes from concrete to abstract smells

### Seven Guiding Principles for Future Children’s Olfactory Picturebooks

#### Avoid Redundance

One of the chief characteristics of high-quality picturebooks is that all elements complement and enrich each other (Nikolajeva & Scott, 2013). Picturebooks that convey the same message in both text and olfaction, or illustration and olfaction, create content redundancy. Future olfactory picturebooks could use olfaction to signal, and not represent, a specific characteristic of the story character or story-plot. For example, to represent mischief, the story page could be sprayed with fresh odours (such as peppermint, rosemary) to convey suspense. To convey that a character has a warm personality, the picturebook page could be enhanced with for example, a cinnamon odour, without the need to refer to cinnamon or warmth in the story text/illustrations. The choice of specific odours will depend on the authors’ intent and target audience, but it should be guided by the aim to integrate olfaction with the story, rather than include it as an add-on.

#### Set Scenes with Ambient Scents

In current picturebooks, the dialogue occurred directly between the story characters, with the smell at the centre of attention. In future, ambient smells can be usefully employed to open or close a scene or afford drama to an implied dialogue. For example, releasing the pine tree aroma during the reading of a digital picturebook could be used to set a scene and add background to the location of the story. Ambient smells can communicate story-relevant meanings that can be implied from dialogues and adding, for example, galbanum scent (very intense turpentine and earthy odour) to a page could signal strength that carries the story forward. In these examples, olfaction is used in a similar way to background music that is often used for introducing or transitioning story scenes (Ziv & Goshen, 2006).

#### Convey Complexity of Characters

The addition of odours to children’s picturebooks could enable the creation of richer and more complex characters. Professional perfume-makers follow a sophisticated classification system of four main categories: Freshness, Air, Earthy, and Fire. In perfumery, the four categories are combined to create perfumes that convey multi-faceted personalities. For example, a “fresh personality” is typically matched with a lively, natural scent of summer flowers such as lilac, freesia, or peony, citrus fruits such as melons and pineapple, and/or marine scents (Delacourte, 2022). In children’s picturebooks, a fresh personality character might be described as speaking in high-pitched voice or depicted in bright colours, and these characteristics could be amplified with corresponding aromas.

#### Customise Story Plots

Olfaction could usefully advance narrative arcs and individual scenes in various directions, based on the scenes and their contents. Essential oils, which are based on concentrated and distilled essences of herbs and plants, can convey short and intense olfactory experiences and could be employed to stimulate readers’ emotions elicited through various scenes in the picturebooks. Uplifting scenes can be created with basil, ginger, or black pepper oils while comforting and soothing scenes can be achieved through a combination of neroli, rose, or magnolia oils. The contrast in olfactory perception could be used to switch, break up, or reverse the order in-between individual pages in the picturebook. Readers could be offered the possibility to mix and match the sequence of stories in a series, thus connecting to their agency in reading for pleasure (Cremin, 2020).

#### Calibrate Olfactory Perceptions

Just as digital books allow for customisation of experience through adjustment of lighting, intensity of colours, or music volume, future digital olfactory books could allow readers’ customisation of odour intensities. The German standard Olfactometry Determination of Odour Intensity can be used for guiding the development and use of odour intensity from 0 = non-perceptible to 6 = extremely strong. Children’s age should be considered when thinking about the concentration of individual ingredients in an odour, given the known variations in odour thresholds, with younger children having higher detection and discrimination abilities of odours compared to adults (Oleszkiewicz et al., 2019). While print olfactory books are limited in their possibility for customisation, digital books might be better suited to different odour intensities required by adult and child readers. Given that smells are only detectable when they are airborne, olfactory perceptions should be considered in relation to individual children’s health conditions (e.g., respiratory illnesses).

#### Use Scents for Immersion

Picturebooks offer an important arena for children’s practice of perspective-taking, that is the ability to detect and interpret the mental states of others, and odour could be used to stimulate readers’ theory of mind and empathy with story characters. For example, a combination of jasmine and lavender scents would convey a calming, sleeping mood, which may support readers to better relate to a tired story character depicted in the book.

Readers carry diverse body odours and diverse olfactory memories: ‘Scents are subliminally immersive; we readily become part of the invisible scent of a landscape. When we inhale a scent, its chemical composition becomes part of us; it passes through our bodies and returns changed into the environment’ (p. 75, Lauriault & Lindgaard, 2006). Authors of olfactory books could capitalise on the variety of odours brought about by individual readers and actively evoke personal memories through odour-related story prompts (in text, pictures, or physical smell stimulation). In such a process it would be important to establish differences between positive olfactory memories that can facilitate story immersion and negative olfactory associations that could recall aversive memories and traumatic experiences (see Toffolo et al., 2012).

#### Transition from Concrete to Abstract Smells

The change from one literary genre to another occurred in Sipe’s (2008) study in relation to page breaks in *Where the Wild Things Are* (Sendak, 1963). Similar change could be achieved in olfactory picturebooks through a change from concrete to abstract smells. Abstract smells are more likely to stimulate children’s fantasy, wonder, and different time constellations, for example, traveling in time to future or back in history. The relative importance of concrete versus abstract characteristics in complex odour compositions can also contribute to metaphorical representations. Lee and Schwarz (2012) have discussed the representational nature and linguistic consequences of abstract and concrete smells in relation to metaphor theories. They highlighted that when the progress from concrete to abstract smells is facilitated through odours, it matches natural developmental progress, which can be harnessed in metaphorical mappings in cognition. While the level of abstraction in olfactory language varies across cultures (see Majid et al., 2018), there are also some common patterns across cultures, such as the progression from concrete to abstract sensorimotor experiences (Lee & Schwarz, 2012). It follows that the sequence from concrete to abstract smells in future children’s olfactory picturebooks would be in alignment with the literary guide on metaphorical representations.

## Limitations of Olfactory Books and of Our Review

Our review of current olfactory books was limited to the Amazon website and the English language. On one hand, it allowed us to get a convenient overview of books popular in several English-speaking countries but on the other hand, it did not allow for an exploration of cultural nuances and non-English picturebooks. Future studies could extend our observations with an overview of olfactory picturebooks in different languages and in relation to the tradition of children’s literature in different countries.

There is a danger that in an effort to capture young children’s interest in reading, olfaction becomes a hook without offering the literary experience one might expect from a children’s picturebook. Our guiding principles for future children’s olfactory picturebooks are a suggested way to address the current limitations of popular children’s olfactory books, with a specific focus on the olfactory element and its role in story. The literary quality of the picturebook as a whole, depends, however, not only on the integration of olfaction with the story but crucially, on the overall quality of writing (in terms of word choice, grammatical complexity) and aesthetic quality of the illustrations. Future research could perform an analysis of the extent to which olfactory picturebooks vary in the quality (sophistication) of text and how this relates to the integration of olfaction in individual titles.

A clear limitation of olfactory books is the emphasis on engaging a sense that might be dysfunctional or temporary disabled in children suffering from respiratory diseases, including asthma, chronic cough, or laryngopharyngitis. Furthermore, given that some of the scents used in the books might contain volatile organic compounds (compounds that are also found in other fragranced products, see Steinemann, 2018), children might have an adverse reaction and health effects. Thus, while olfactory picturebooks might provide new sensory stimulation for children with visual or hearing impairments, they restrict the stimulation for children with olfactory impairment.

Future research and design of olfactory picturebooks should consider inclusive approaches that enable the engagement of multiple senses, with olfaction being one, but not the exclusive, way to enter and engage with a story. An inclusion lens on olfactory picturebooks should also bring to fore the cross-cultural differences in olfactory preferences and representations in the society and the political messaging around some odours perceived as undesirable or pertaining to lower socio-economic groups. In this respect, olfactory picturebooks have the potential to challenge and broaden not only the literary but also socio-cultural aspects of children’s reading.

## Conclusion

Our short “tour” of current children’s olfactory picturebooks is an attempt to sketch their potential for children’s literary studies. Building on Sipe’s (2008) theorisations of how children can learn from illustrations in picturebooks, we propose a departure from the current use of olfaction as an add-on feature in print and scratch-and-sniff books, to a more integrated experience blending pictures and stories. Instead of focusing on only bad and good smells that reflect binary qualities of stories, olfaction could deliver sophisticated literary qualities, including scene-setting, increasing story suspense, and readers’ immersion.

Traditional picturebook theories have emphasised the power of images in holding steer and control of the narrative art in children’s picturebooks. Nodelman (1988, p. 101) wrote that pictures carry “visual weight” and that most narrative information comes from objects depicted in pictures. We suggest that similar narrative information can be created with olfactory features. This proposition carries theoretical implications, as it contests and subverts the dominant visual focus of children’s literacies, which has been criticised for socio-historical biases that lie at the root of inequitable learning in early years classrooms (see Hackett et al., 2015; Ehret & Rowsell, 2021; Lee et al., 2022).

As reading is becoming more hybrid and more transmedia, digital olfactory books could offer fruitful insights into established perceptions of what a children’s picturebook conveys or looks like. Overall, the diverse possibilities for integrating olfaction into picturebooks stimulate opportunities for innovation in both the format and content of future children’s literacies.
